# Analyses of the Effects of Wild‐Type TDP‐43 Overexpression in Oxytocin Neurons in Mice

**DOI:** 10.1111/nan.70059

**Published:** 2026-01-21

**Authors:** Sofia Bergh, Oskar Simonsson, Åsa Petersén

**Affiliations:** ^1^ Translational Neuroendocrine Research Unit, Department of Experimental Medical Science Lund University Lund Sweden; ^2^ Department of Psychiatry Skåne University Hospital Lund Sweden

**Keywords:** amyotrophic lateral sclerosis, frontotemporal dementia, hypothalamus, metabolism, oxytocin, TDP‐43

## Abstract

- Selective TDP‐43 overexpression in oxytocin neurons in the paraventricular nucleus of the hypothalamus causes a decrease in oxytocin‐immunopositive cells compared to uninjected mice.
- AAV‐mediated TDP‐43 overexpression in oxytocin neurons does not appear to be a major driver of behavioural and metabolic phenotypes in mice.

**Abbreviations**
AAV: adeno‐associated virus
AAV‐GFP: AAV‐vector expressing GFP
AAV‐TDP43: AAV‐vector expressing TDP‐43
ALS: amyotrophic lateral sclerosis
ANOVA: analyses of variance
Cre: Cre‐recombinase
DEXA: dual‐energy x‐ray absorptiometry
EPM: elevated plus maze
FST: forced swim test
FTD: frontal temporal dementia
GFP: green fluorescent protein
MCH: melanin‐concentrating hormone
Oxytocin‐Cre: mice expressing Cre‐recombinase under the oxytocin promoter
PVN: paraventricular nucleus
TDP‐43: TAR DNA‐binding protein 43

TAR DNA‐binding protein 43 (TDP‐43) is the major pathological protein in amyotrophic lateral sclerosis (ALS) [1]. Motor symptoms in ALS are often preceded by psychiatric features, including apathy and depression, as well as metabolic dysfunction characterised by a reduced body mass index [2, 3]. The hypothalamus, a key regulator of metabolism and autonomic and endocrine responses to emotion, is affected in clinical ALS [4]. Recent post‐mortem studies on ALS cases have revealed TDP‐43 inclusions in and atrophy of the hypothalamus, along with the selective loss of key hypothalamic neuronal populations that express oxytocin, hypocretin (orexin) or melanin‐concentrating hormone (MCH). In addition, TDP‐43 inclusions are found in oxytocin neurons in ALS cases [5, 6, 7]. Because oxytocin is a neuropeptide important for complex social behaviour, metabolism, anxiety and depression, its pathology may be linked to the emotional and metabolic dysregulation seen early in ALS [8]. Also, some effects on apathy have been found after administration of oxytocin to patients with another TDP‐43 proteinopathy, frontal temporal dementia (FTD) [9]. A recent study demonstrated that overexpression of wild‐type TDP‐43 in the entire hypothalamus of mice replicates clinical hypothalamic pathology in ALS, including loss of neurons expressing oxytocin, hypocretin and MCH as well as the development of metabolic dysfunction and apathy [10]. However, the cell‐autonomous effect of overexpression of wild‐type TDP‐43 in oxytocin neurons on neuropathology, metabolism and behaviour remains to be investigated. Understanding this direct effect could be important for understanding the role of the oxytocin system in neurodegenerative disorders with TDP‐43 pathology. In the present study, we therefore aimed to investigate whether selective overexpression of wild‐type TDP‐43 in oxytocin neurons would have effects on neuropathology as well as metabolic and behavioural features.

We used a flex‐switch adeno‐associated viral (AAV) vector to express either wild‐type human TDP‐43 (AAV‐TDP43) or GFP (AAV‐GFP) in oxytocin neurons in mice. Transgene expression was dependent on the presence of Cre‐recombinase (Cre), which was expressed under the oxytocin promoter in mice (oxytocin‐Cre mice; B6; 129S‐Oxt^tm1.1(Cre)Dolsn^/J, Jackson Laboratories). Flex‐switch AAV‐vectors (titre: 2.0 E14 genome copies/mL) were bilaterally injected into the paraventricular nucleus (PVN) of the hypothalamus of 6‐week‐old oxytocin‐Cre mice. Housing conditions, AAV vector production and stereotactic delivery were similar to those described previously [11].

For neuropathological analyses, mice were perfused at 24 weeks post‐injection. The cohort included 11 AAV‐TDP43 (female = 7, male = 4) or 7 AAV‐GFP (female = 5, male = 2) injected mice and 9 age‐matched uninjected controls (female = 9). One AAV‐GFP and one AAV‐TDP43 brain were excluded from neuropathological analyses due to unsuccessful sectioning. Tissue preparation and immunohistochemical analyses as well as image acquisition and processing were performed as previously described [10, 11]. For vector targeting and quantification of oxytocin‐expressing neurons, images of coronal sections between bregma −0.59 to −0.94 were acquired at 10×/0.25 NA using a ZEISS Primostar microscope and analysed in ImageJ 1.53t software. The oxytocin‐expressing cells were quantified manually using ImageJ's multipoint tool, whereas the cells overexpressing TDP‐43 were quantified with an automated ImageJ script thresholding for pixel intensity (0–100) and circularity (0.30–1.00). Missing sections were imputed with an average from the same group and bregma. The total number of neurons was estimated by multiplying the observed cell count by the cutting interval of the collected sections (i.e., every sixth section was counted, resulting in a multiplier of 6).

Effects on the behavioural and metabolic phenotype following TDP‐43 overexpression in oxytocin neurons were investigated in the same cohort of mice with 11 AAV‐TDP43 (female = 7, male = 4) and 8 AAV‐GFP (female = 5, male = 3) injected oxytocin‐Cre mice. Behavioural experiments were performed at 20–23 weeks post‐injection as previously described [10, 11]. These tests included elevated plus maze (anxiety‐like behaviour), forced swim test (depressive‐like behaviour), social interaction test (social cognition) and rotarod (motor coordination and motivation). Body weight measurements and dual‐energy x‐ray absorptiometry (DEXA) scans were performed at 23 weeks post‐injection.

Statistical analyses were performed in SPSS 29.0. One‐way ANOVA with Tukey's post hoc was used for normally distributed data, whereas the Mann–Whitney *U* test and Spearman's ranks correlation were used for non‐parametric data. A *p*‐value of < 0.05 was considered significant.

Immunohistochemical analyses confirmed that TDP‐43 and GFP immunoreactivity were limited to the PVN of oxytocin‐Cre mice (Figure 1A–C). Immunofluorescent analysis confirmed that TDP‐43 overexpression was selective to oxytocin neurons (Figure 1D). The flex‐switch AAV‐GFP vector has been previously validated [11]. The AAV‐TDP43 vector achieved a targeting efficacy of around 60% (range 13%–91%) of oxytocin neurons in the PVN (Figure 1J). A trend towards a negative correlation was found between the number of TDP‐43‐overexpressing cells and the number of oxytocin‐expressing cells (*r*(8)= − 0.61, *p* = 0.060; Figure 1K).

**FIGURE 1:**
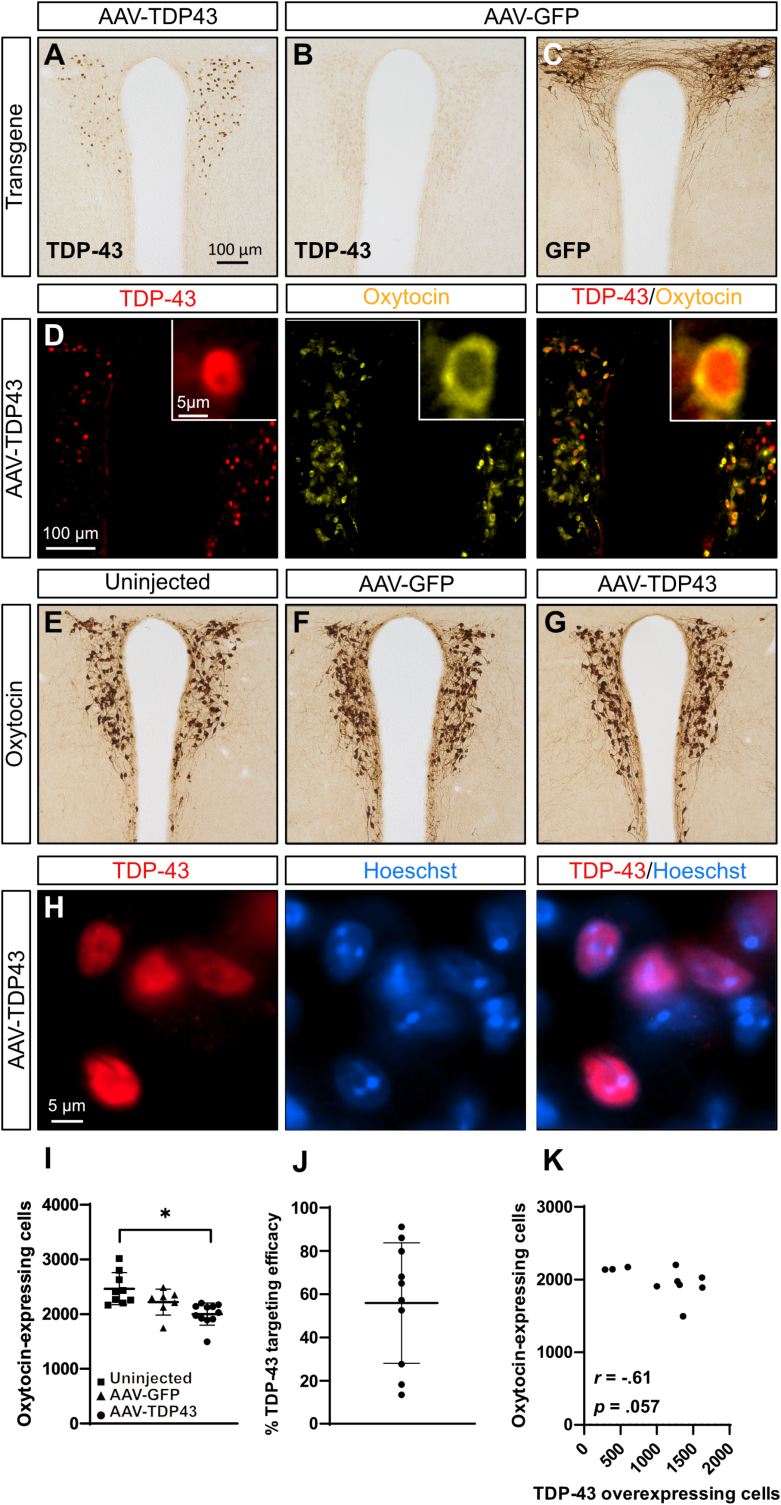
Selective TDP‐43 overexpression in oxytocin neurons results in loss of oxytocin‐expressing neurons compared to uninjected mice. Oxytocin‐Cre mice were injected bilaterally with either an AAV‐TDP43 or AAV‐GFP vector, resulting in selective transgene expression in oxytocin neurons. (A–C) At 24 weeks post‐injection, TDP‐43 and GFP immunoreactivity were detected, limited to the PVN. (D) TDP‐43 overexpression was found in oxytocin‐immunopositive neurons. (E–G,I) The number of oxytocin‐expressing neurons was reduced in AAV‐TDP43 mice compared to uninjected controls (*p* = 0.001), but not compared to AAV‐GFP mice. (H) Immunofluorescent staining for TDP‐43 and Hoechst (nuclear counterstain) revealed TDP‐43 immunoreactivity limited to the nucleus, without inclusion formation. (J) Stereotactic injections of AAV‐TDP43 led to clear overexpression of TDP‐43 in an average of 60% of oxytocin neurons in the PVN. (K) A trend towards a negative correlation was observed between the number of TDP‐43‐overexpressing cells and the number of oxytocin‐expressing cells (*r*(8)= − 0.61, *p* = 0.060). Data are presented as mean ± SD Statistical analyses: one‐way ANOVA with Tukey's post hoc (I) and Spearman's rank correlation (K). *p < 0.05.

To investigate effects on neuropathology, we performed quantitative analyses of the number of oxytocin‐expressing neurons. AAV‐TDP43‐injected mice displayed a 19% reduction in oxytocin‐expressing neurons (2000 ± 202) compared to uninjected controls (2465 ± 294, *p =* 0.001; Figure 1E–G,I). However, this reduction was not significantly different from AAV‐GFP mice (2217 ± 236, *p = *0.177). There was no difference between uninjected and AAV‐GFP mice (*p = *0.132). This suggests that wild‐type TDP‐43 overexpression can exert mild cell‐autonomous toxicity in oxytocin neurons.

Nuclear‐to‐cytoplasmic mislocalisation and inclusions of TDP‐43 are found in cases with ALS [12, 13] and in our previous pan‐hypothalamic TDP‐43 overexpression mouse model [10]. We therefore investigated the molecular properties of AAV‐mediated TDP‐43 overexpression in oxytocin neurons. TDP‐43 was detected within the nucleus and did not appear to form nuclear or cytoplasmic inclusions in the remaining cells in this model (Figure 1H). Given that the same wild‐type TDP‐43 construct was used as in our previous pan‐hypothalamic TDP‐43 model [10], this finding suggests that oxytocin neurons may be able to manage high levels of wild‐type TDP‐43 within the nucleus without forming inclusions or inducing cytosolic mislocalisation.

Oxytocin plays a role in anxiety, depression, apathy and complex social behaviour, which are early features of ALS [8] as well as in FTD [9]. We therefore investigated the effects of TDP‐43 overexpression in oxytocin neurons on the development of behavioural features. Mice overexpressing TDP‐43 in oxytocin neurons spent a similar percentage of time on open arms compared to AAV‐GFP control mice in the elevated plus maze (Figure 2A) and time spent immobile in the forced swim test (Figure 2B), suggesting no anxiety‐ or depressive‐like phenotypes. The assessment of social behaviour using the social interaction test revealed no significant differences in social affiliation or social novelty (Figure 2C,D) between the different groups. We found no correlation between the results from the behavioural experiments and either the number of oxytocin‐expressing neurons or the number of TDP‐43‐immunopositive cells (data not shown). The AAV‐TDP43‐injected mice had an increased latency to fall as assessed with the rotarod test compared to AAV‐GFP‐injected mice (*p = *0.001; Figure 2E).

**FIGURE 2:**
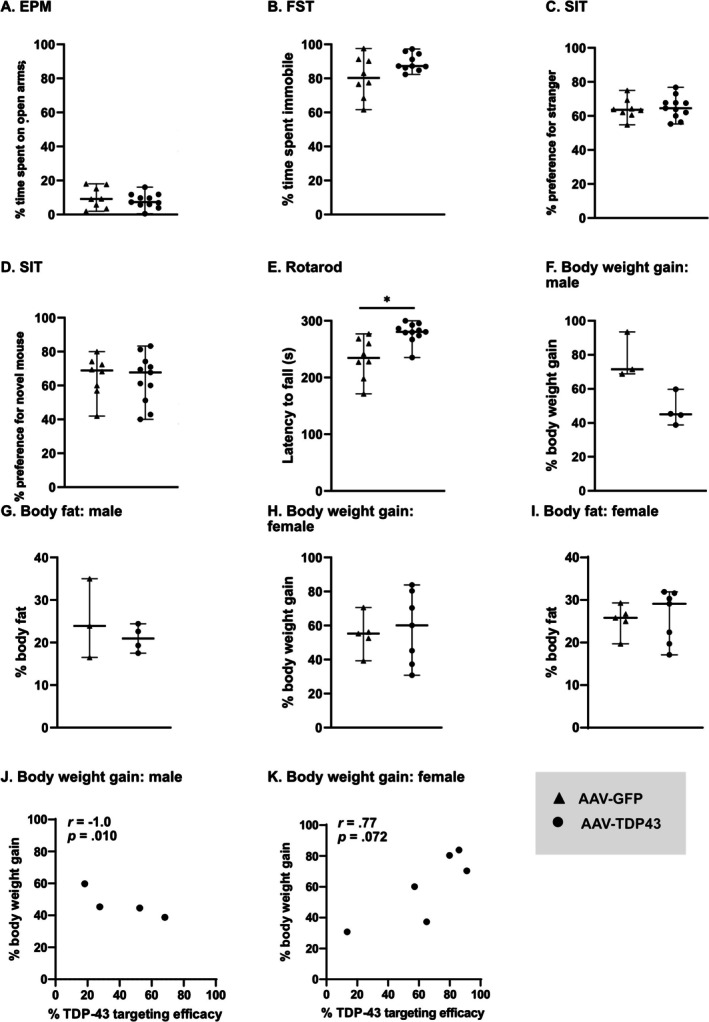
Overexpression of TDP‐43 in oxytocin neurons has no major impact on behavioural or metabolic phenotypes in mice. Behavioural and metabolic assessments were performed at 20–23 weeks post‐injection. (A,B) No significant differences were observed with elevated plus maze (EPM) or the forced swim test (FST). (C,D) The social interaction test (SIT) revealed no differences in social affiliation or social novelty. (E) AAV‐TDP43 mice displayed a significantly increased latency to fall in the rotarod test compared to AAV‐GFP mice (*p* = 0.001). (F–I) No significant differences were found in body fat percentage or body weight change across sex and treatment groups; however, there was a trend of decreased body weight gain in male AAV‐TDP43 mice (*p* = 0.057). (J) A strong negative correlation was found in male mice between the TDP‐43 targeting efficacy and body weight gain. (K) Female mice displayed a trend towards a positive correlation between TDP‐43 targeting efficacy and body weight gain. Data are presented as median with range. Statistical analyses: Mann–Whitney *U* test (A–I) and Spearman's rank correlation (J–L).

Because hypothalamic atrophy has been associated with reduced body mass index preceding motor symptoms in ALS [14], we investigated the metabolic phenotype in mice overexpressing TDP‐43 in oxytocin neurons. We observed no effect on body weight gain and body fat percentage, regardless of sex and treatment (Figure 2F–I). However, we observed a trend of decreased body weight gain in male AAV‐TDP43 mice compared to male AAV‐GFP mice (*p =* 0.057; Figure 2F).

Given the observed variability of TDP‐43 targeting efficacy in oxytocin neurons, we performed Spearman's rank correlation analyses between TDP‐43 overexpression targeting efficacy and metabolic and behavioural features. A strong negative correlation was detected in male mice between TDP‐43 targeting efficacy and body weight gain (*r*(4)= − 1.00, *p* = 0.01; Figure 2J). Conversely, the analysis in female mice showed a trend that did not reach statistical significance towards a positive correlation between TDP‐43 expression efficacy and body weight gain (*r*(6) = 0.77, *p* = 0.072; Figure 2K). These differences between males and females suggest that TDP‐43 may interact with sex‐specific metabolic pathways in oxytocin neurons. A recognised limitation of using AAV‐mediated expression is the inherent variability in targeting efficacy; however, we did not detect any other significant correlations between the TDP‐43 targeting efficacy and the remaining phenotypic features measured (Figure S1). Taken together, these data suggest that overexpression of wild‐type TDP‐43 in oxytocin neurons has limited effects on the development of behavioural and metabolic features.

This study reveals a mild cell‐autonomous neurotoxic effect of overexpressed wild‐type TDP‐43 on oxytocin neurons. We observed a decrease in the number of neurons expressing oxytocin compared to uninjected controls, but not relative to AAV‐GFP mice. In our recent study [12], pan‐hypothalamic overexpression of wild‐type TDP‐43 resulted in a 42% loss of oxytocin neurons, downregulation of oxytocin transcripts and clear metabolic and behavioural impairment. The difference in oxytocin system pathology between the two models may imply that the effect on oxytocin is not solely cell autonomous. Our data also suggest that metabolic and behavioural phenotypes observed after overexpression of TDP‐43 in the hypothalamus are likely not a result of TDP‐43 dysfunction in oxytocin neurons. Hence, further studies investigating the effects of TDP‐43 on other hypothalamic neuronal populations may shed light on the relationship between specific hypothalamic circuitry dysfunction and the development of non‐motor manifestations in TDP‐43 proteinopathies.

## Author Contributions

All authors contributed to the study conception and design. Material preparation, data collection and data analysis were performed by all authors. The first draft of the manuscript was written by Sofia Bergh, Oskar Simonsson and Åsa Petersén. All authors read and commented on previous versions of the manuscript. All authors read and approved the final manuscript.

## Funding

This study was funded by research grants to Åsa Petersén from the Swedish Research Council (grant number 2022/01092), the Swedish Brain Foundation, the Swedish governmental funding of clinical research (ALF) at Region Skåne and the Knut and Alice Wallenberg Foundation (# 2019.0467).

## Ethics Statement

All animal procedures within this project have been approved by the Malmö/Lund ethical committee according to permit number 17113/2022.

## Conflicts of Interest

The authors declare no conflicts of interest.

## Supporting information


**Figure S1:** Correlation analysis of TDP‐43 targeting efficacy and phenotypic outcomes in mice. Spearman's rank correlation analyses examining the relationship between the percentage of TDP‐43 expression oxytocin neurons and results from (A) elevated plus maze (EPM), (B) forced swim test (FST), (C,D) social interaction test (SIT), (E) rotarod, body fat percentage in male (F) and female (G) mice.

**Data S1:** Supporting Information.

## Data Availability

The data that support the findings of this study are available from the corresponding author upon reasonable request.
