# Supplementary figures and images for: Analyses of the Effects of Wild‐Type TDP‐43 Overexpression in Oxytocin Neurons in Mice
Source: Neuropathol Appl Neurobiol. 2026 Jan 21;52(1):e70059. doi: 10.1111/nan.70059 (PMC12822521; doi:10.1111/nan.70059)

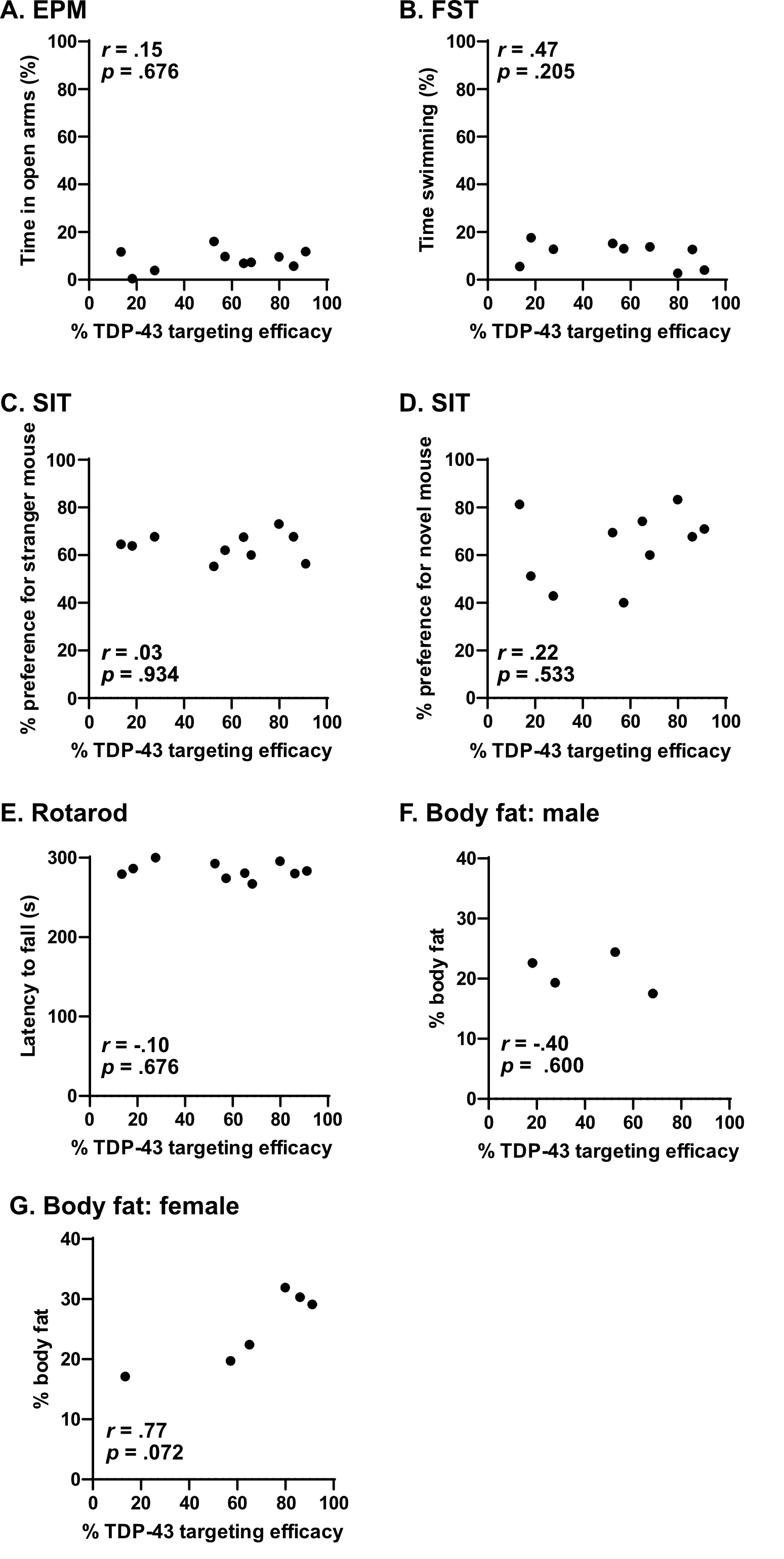

Supplement: Supplementary file 1 — Figure S1: Correlation analysis of TDP‐43 targeting efficacy and phenotypic outcomes in mice. Spearman's rank correlation analyses examining the relationship between the percentage of TDP‐43 expression oxytocin neurons and results from (A) elevated plus maze (EPM), (B) forced swim test (FST), (C,D) social interaction test (SIT), (E) rotarod, body fat percentage in male (F) and female (G) mice. [file NAN-52-e70059-s002.jpg]
