# Supplementary material for: Analyses of the Effects of Wild‐Type TDP‐43 Overexpression in Oxytocin Neurons in Mice
Source: Neuropathol Appl Neurobiol. 2026 Jan 21;52(1):e70059. doi: 10.1111/nan.70059 (PMC12822521; doi:10.1111/nan.70059)
Supplement: Supplementary file 2 — Data S1: Supporting Information. [file NAN-52-e70059-s001.docx]

# **Supplementary material:** **Analyses of the effects of wild-type TDP-43 overexpression in oxytocin neurons in mice**

Sofia Bergh^1*^, Oskar Simonsson^1*^, and Åsa Petersén^1, 2^.

^1^Translational Neuroendocrine Research Unit, Department of Experimental Medical Science, Lund University, 221 84 Lund, Sweden

^2^Department of Psychiatry, Skåne University Hospital, Lund, Sweden

*: Shared first author

**Corresponding author:** Correspondence should be addressed to Sofia Bergh (email: [sofia.bergh@med.lu.se](mailto:sofia.bergh@med.lu.se)), Translational Neuroendocrine Research Unit, Department of Experimental Medical Science, Lund University, BMC D11, 221 84 Lund, Sweden.

## **Supplementary figure 1**


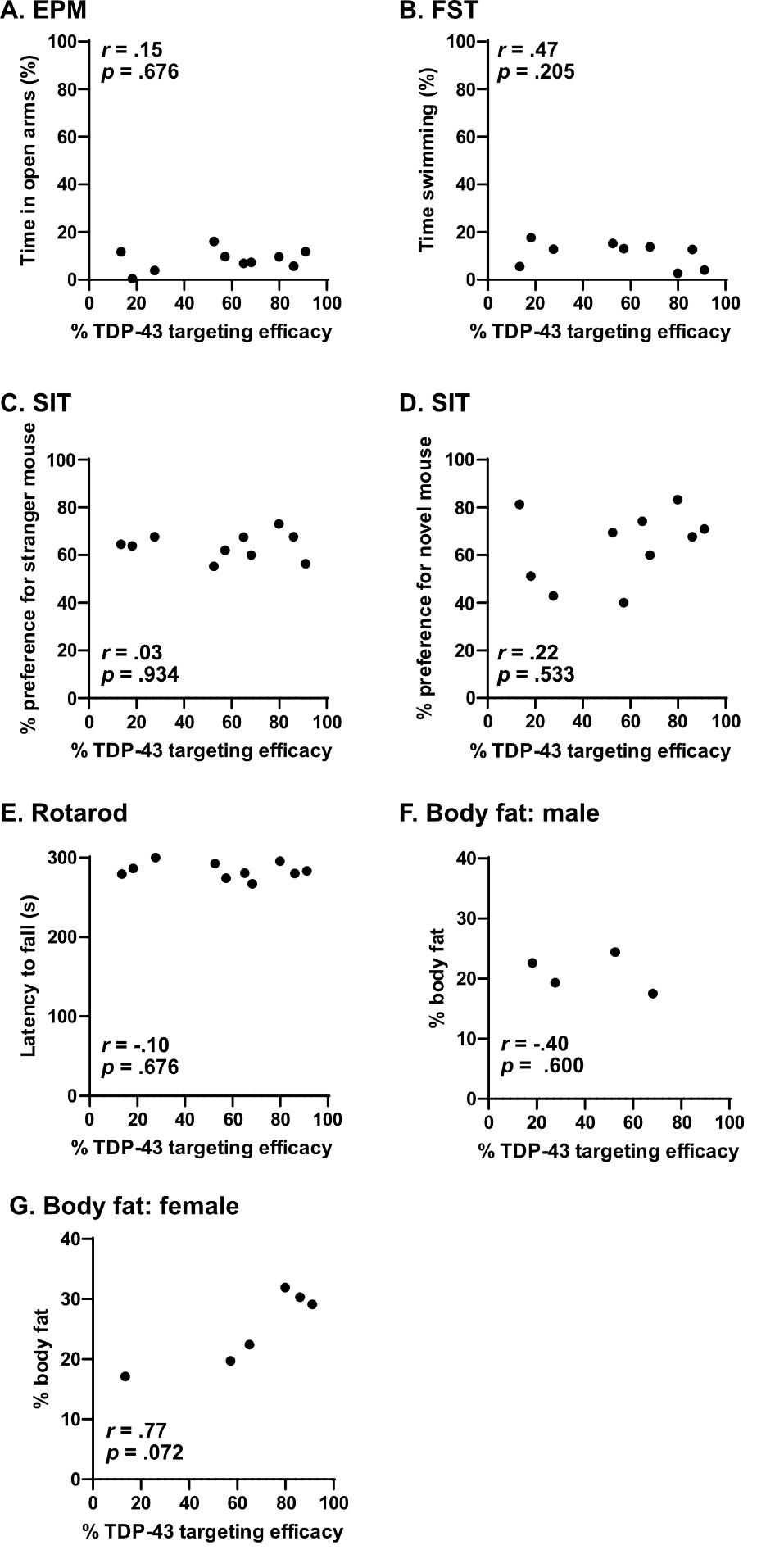


**Supplementary Figure 1. Correlation analysis of TDP-43 targeting efficacy and phenotypic outcomes in mice.** Spearman’s rank correlation analyses examining the relationship between the percentage of TDP-43 expression oxytocin neurons and results from (**A**) elevated plus maze (EPM), (**B**) forced swim test (FST), (**C-D**) social interaction test (SIT), (**E**) rotarod, body fat percentage in male (**F**) and female (**G**) mice.
